# 

**Published:** 1986-12

**Authors:** 


### Contents

125 **Editorial**

126 **Appendicitis in Bristol—100 years ago**

M. J. Cooper

129 **The assessment of the Urological Opprobria**

R. C. L. Feneley

132 **Learning about Haemophilia**

Carol Kendrick

134 **The Bristol Eye Hospital—an historical perspective**

V. J. Marmion

135 **From our Correspondents**

139 **From our Foreign Correspondent. Bus 237 to Pokhara**

J. L. Burton

141 **Glass Paperweights**

F. G. M. Ross

145 **Distraction, displacement and alarm**

Philip Radford

147 **Poet's Corner**

Michael Lennard, John Hughes Games, Michael Wilson

150 **Index for 1986**

